# Hyperglycemia is associated with worse 3-year survival in older patients admitted to the intensive care unit after non-cardiac surgery: *Post hoc* analysis of a randomized trial

**DOI:** 10.3389/fmed.2022.1003186

**Published:** 2022-12-12

**Authors:** Mo Li, Chun-Mei Deng, Xian Su, Dan-Feng Zhang, Mao Ding, Jia-Hui Ma, Dong-Xin Wang

**Affiliations:** ^1^Department of Anesthesiology and Critical Care Medicine, Peking University First Hospital, Beijing, China; ^2^Outcomes Research Consortium, Cleveland, OH, United States

**Keywords:** aged, surgical procedures, operative, intensive care unit, hyperglycemia, long term adverse effects, survival

## Abstract

**Objective:**

Hyperglycemia is common in critically ill patients after surgery and is associated with worse perioperative outcomes. Yet, the impact of postoperative hyperglycemia on long-term outcomes remains unclear. We therefore analyzed the association between early postoperative hyperglycemia and 3-year overall survival in older patients who were admitted to the intensive care unit after surgery.

**Methods:**

This was a *post hoc* analysis of database obtained from a previous randomized trial and 3-year follow-up. The underlying trial enrolled 700 patients aged 65 years or older who were admitted to the intensive care unit after elective non-cardiac surgery. Early postoperative time-weighted average blood glucose was calculated and was divided into three levels, i.e., <8.0 mmol/L, from 8.0 to 10.0 mmol/L, and >10.0 mmol/L. The primary outcome was 3-year overall survival. The association between time-weighted average blood glucose level and 3-year overall survival was analyzed with Cox proportional hazard regression models. Subgroup analyses were also performed in patients with or without diabetes, and in patients following cancer or non-cancer surgery.

**Results:**

A total of 677 patients (mean age 74 years, 60% male sex) were included in the final analysis. Within 3 years after surgery, deaths occurred in 22.1% (30/136) of patients with time-weighted average blood glucose <8.0 mmol/L, compared with 35.7% (81/227) of those from 8.0 to 10.0 mmol/L (unadjusted hazard ratio 1.75, 95% CI 1.15 to 2.67, *P* = 0.009), and 36.9% (116/314) of those >10.0 mmol/L (unadjusted hazard ratio 1.91, 95% CI 1.28 to 2.85, *P* = 0.002). After adjustment for confounding factors, the risk of 3-year mortality remained higher in patients with time-weighted average blood glucose from 8.0 to 10.0 mmol/L (adjusted hazard ratio 2.28, 95% CI 1.47 to 3.54, *P* < 0.001) and in those >10.0 mmol/L (adjusted hazard ratio 2.00, 95% CI 1.29 to 3.10, *P* = 0.002). Similar results were obtained in the subgroups of patients without diabetes and patients following cancer surgery.

**Conclusion:**

For older patients admitted to the intensive care unit after elective non-cardiac surgery, high early blood glucose (time-weighted average blood glucose ≥ 8.0 mmol/L) was associated with poor 3-year overall survival. The impact of moderate glycemic control on long-term survival deserves further investigation.

## Introduction

Hyperglycemia is common in critically ill patients, including those admitted to the intensive care unit (ICU) after surgery (1). Postoperative hyperglycemia occurs in diabetes patients, but may also occur in those without (2–5). The reported incidence ranges from 10.5 to 46.7%, depending on diagnostic criteria and surgical populations (6–8). Multiple factors contribute to postoperative hyperglycemia and may include comorbid diabetes, severity of acute illness, surgical stress, and new-onset complications (9).

Early postoperative hyperglycemia is associated with adverse clinical outcomes including increases in postoperative complications (6, 10–13), length of hospital stay (6, 11), medical expenses (11, 13), and perioperative mortality (6, 7, 9). As a matter of fact, persistent postoperative hyperglycemia has been reported to be a useful predictor of complications that require readmission and reoperation (7, 14). The association between early hyperglycemia and high in-hospital mortality is also reported in patients with other critical illnesses, such as myocardial infarction, respiratory failure, stroke, sepsis, and trauma (9, 15–18), and is especially true in patients without diabetes (6, 9, 19). On the other hand, a recent meta-analysis showed that intensive glucose control in critically ill patients reduces acquired infection and sepsis, length of ICU stay, and all-cause mortality, although it increases hypoglycemic events (20); this further indicates the importance of monitoring and managing blood glucose.

Evidence regarding the impact of postoperative hyperglycemia on long-term outcomes is limited. In a retrospective study of 505 patients following peripheral arterial bypass surgery, immediate postoperative hyperglycemia (≥7.8 mmol/L) was associated with an increased risk of long-term amputations (13). In another retrospective study, 104 patients were included in the analysis after propensity score matching; those with postoperative hyperglycemia (≥ 7.8 mmol/L) had worse long-term survival after gastric cancer surgery (21). Whereas a 44-month follow-up of 189 patients enrolled in a randomized trial showed that strict (5–6.7 mmol/L) compared with liberal (6.7–10 mmol/L) glucose management did not change long-term survival and quality of life after coronary bypass surgery (22), but the trial was seriously underpowered. Currently, there is no consensus regarding the long-term effect of postoperative hyperglycemia and the optimal glucose level based on long-term outcomes following surgery.

In a previous trial, we enrolled 700 patients aged 65 years or older who were admitted to the ICU after elective non-cardiac surgery and investigated the impact of prophylactic low-dose dexmedetomidine on delirium (23). We then performed a 3-year follow-up of these patients and evaluated the long-term outcomes including overall survival, quality of life, and cognitive function (24). The objective of this *post hoc* analysis was to explore the relationship between early postoperative blood glucose and 3-year survival in these patients.

## Materials and methods

### Study design

This was a *post hoc* analysis of the database from a randomized trial and 3-year follow-up. The protocols of the underlying studies were approved by the local Clinical Research Ethics Committee (2011 [10] and 2014 [711]), registered with the Chinese Clinical Trial Registry (ChiCTR-TRC-10000802) and ClinicalTrials.gov (NCT02809937), and published previously (23, 24). This analysis was performed in compliance with the Declaration of Helsinki and approved by the Biomedical Research Ethics Committee of the Peking University First Hospital, Beijing, China (2022 [074]). Informed consent was waived by the Ethics Committee because all data were obtained from the available database and the inpatient medical record system and no patient-contact or family member-contact was needed. However, personal data were kept strictly confidential.

### Participant recruitment

In the underlying trial, we enrolled patients aged ≥65 years who were admitted to the ICU after elective non-cardiac surgery under general anesthesia. We excluded those who met any of the following criteria: (1) preoperative history of schizophrenia, epilepsy, Parkinson’s disease, or myasthenia gravis; (2) inability to communicate because of coma, severe dementia, or language barriers before surgery; (3) brain trauma or neurosurgery; (4) preoperative left ventricular ejection fraction <30%, sick sinus syndrome, heart rate <50 beats/min, or two degrees or higher atrioventricular block without pacemaker; (5) severe liver dysfunction (Child-Pugh C grade) or severe renal dysfunction (preoperative renal replacement therapy); or (6) expected survival ≤24 h (23). For the purpose of this *post hoc* analysis, we also excluded patients with less than 2 blood glucose tests from ICU admission to the next 10 am in the medical records.

### Anesthesia and perioperative care

Intraoperative monitoring was performed according to the American Society of Anesthesiologists (ASA) guidelines and local routine, including intra-arterial pressure and central venous pressure when necessary. Dexamethasone (5–10 mg) was administered before anesthesia induction to prevent postoperative nausea and vomiting. General anesthesia was induced with midazolam and propofol and/or etomidate, and maintained with propofol infusion and/or sevoflurane inhalation, with or without epidural block and/or nitrous oxide inhalation. Sufentanil and/or remifentanil were administered for intraoperative analgesia; neuromuscular blocking agents were used for muscle relaxation. Anesthesia depth was monitored according to clinical signs and bispectral index if available. Blood products were transfused to maintain hemoglobin ≥7 g/dL. The target was to maintain blood pressure within 30% of baseline, nasopharyngeal temperature ≥36°C, and urine output ≥0.5 ml/kg/h.

After ICU admission, all patients were routinely monitored and usually included intra-arterial pressure. Patient-controlled intravenous or epidural analgesia was routinely provided. Supplemental morphine and/or non-steroid anti-inflammatory drugs were administered when necessary. For those with endotracheal intubation, propofol and/or midazolam were administered for sedation; the target was to maintain a Richmond Agitation Sedation Scale [score ranges from –5 (unarousable) to + 4 (combative) and 0 indicates alert and calm (25)] between –2 and +1. Early extubation was encouraged whenever possible. Daily sedation interruption was performed for those who were not extubated the next morning. As the randomized intervention of the underlying trial, either low-dose dexmedetomidine (0.1 μg/kg/h) or normal saline was infused from ICU admission until 8 am on postoperative day 1 (23).

As a routine practice, all patients had their first blood glucose tested immediately on ICU admission. Blood glucose values were obtained from arterial blood gas results (ABL 800 FLEX, Radiometer Medical ApS, Denmark), which were then tested at 4 to 6-h intervals until ICU discharge. The frequency of blood glucose monitoring was increased if abnormal values appeared. Blood glucose management during the ICU stay was standardized in our institution, i.e., regular insulin was added to glucose-containing infusion solution at a ratio of 1:6 for patients without diabetes or 1:4 for patients with diabetes. Additional insulin was injected intravenously when hyperglycemia occurred; specifically, 4 units insulin when blood glucose >10.0 mmol/L, 6 units when >12.0 mmol/L, and 8 units when >15.0 mmol/L. Blood glucose was rechecked every hour and managed in the same way. In case that blood glucose remained >10.0 mmol/L after 2 to 3 bolus injections, insulin was infused continuously, and the glucose infusion rate was reduced when necessary. In case that blood glucose was <5.0 mmol/L, 10 to 20 mL of 50% glucose was injected intravenously, and the glucose/insulin infusion rate was adjusted. The target was to maintain blood glucose between 5.0 and 10.0 mmol/L. ICU discharge was decided by intensivists and surgeons.

### Baseline and perioperative data

Baseline data included age, sex, body mass index, preoperative comorbidities and medication, duration and treatment of diabetes mellitus, and main preoperative laboratory test results. General status was evaluated and included the ASA classification, Charlson Comorbidity Index (26), and Barthel Index (27). The pathological diagnoses were recorded; the tumor-node-metastasis stage for patients following cancer surgery was evaluated according to the American Joint Committee on Cancer 8th Edition Cancer Staging System (28).

Intraoperative data included the type and duration of anesthesia, intraoperative medication, intraoperative fluid infusion and blood product transfusion, estimated blood loss, and the site and duration of surgery. The level of operative stress was rated according to the Operative Stress Score, which stratified physiologic stress into 5 categories, i.e., very low stress, low stress, moderate stress, high stress, and very high stress (29).

Postoperative data included the worst Acute Physiology and Chronic Health Evaluation II (APACHE II) score within 24 h after ICU admission, use and type of patient-controlled analgesia, the occurrence of delirium within seven days, the occurrence of non-delirium complications within 30 days, and lengths of stay in the ICU and hospital after surgery. Delirium was assessed twice daily (from 8 to 10 am and from 18 to 20 pm) with the Confusion Assessment Methods for the Intensive Care Unit (30, 31); non-delirium complications were defined as new-onset medical events other than delirium that required therapeutic intervention (23). For the purpose of this study, we collected in detail the blood glucose values from ICU admission until 10 am next day from the inpatient medical record system. Hypoglycemia was defined as a blood glucose level below 3.9 mmol/L. Severe hypoglycemia was defined as a blood glucose level below 2.2 mmol/L.

### Three-year follow-up data

The 3-year follow-ups were performed by contacting patients and/or their family members via telephone during the underlying study. Cognitive function was assessed with the modified Telephone Interview for Cognitive Status (TICS-m). This is a 12-item questionnaire that evaluates global cognitive function via telephone; the score ranges from 0 to 50, with a higher score indicating better function (32). Quality of life was assessed with the World Health Organization Quality of Life brief version (WHOQOL-BREF). This is a 24-item questionnaire that evaluates physical, psychological, social relationship, and environmental domains; for each domain, the score ranges from 0 to 100, with a higher score indicating better function (33). For patients who died within 3 years, the exact date of death was recorded. For those who were lost to follow-up, the time of last contact or hospital visit after surgery was considered as censoring time.

The primary outcome of this analysis was 3-year overall survival after surgery. Secondary outcomes included cognitive function and quality of life in 3-year survivors. Exploratory analyses were performed in the subgroups of patients with or without diabetes, depending on history of diabetes mellitus, and patients following cancer or non-cancer surgery, depending on the pathological examination results.

### Statistical analysis

#### Time-weighted average blood glucose

To adequately reflect the time exposed to various blood glucose levels and minimize bias caused by irregular measurement intervals, we calculated the time-weighted average (TWA) blood glucose concentration for each patient from ICU admission to the next 10 am on postoperative day 1. TWA blood glucose was calculated as the area under the curve divided by the time interval between the first and the last measurement in two steps (34). First, we calculated the area under the curve for blood glucose values greater than zero using the following formula: area under curve = [(X_1_ + X_2_) (T_2_ – T_1_) + (X_2_ + X_3_) (T_3_ – T_2_) + (X_3_ + X_4_) (T_4_ – T_3_) + … + (X _n_–1 + X _n_) (T_n_ – T_n–1_)]/2; in the equation, X1 is the first glucose value which was measured at time T_1_, and X _*n*_ is the last glucose value which was measured at time T_n_. Second, TWA blood glucose = area under curve/(T_*n*_-T_1_).

#### Outcome analysis

Patients were arbitrarily divided into three groups based on the TWA blood glucose concentration, i.e., those <8.0 mmol/L, those from 8.0 to 10.0 mmol/L, and those >10.0 mmol/L. Continuous variables with normal distribution were presented as mean ± SD and analyzed using one-way ANOVA followed by Bonferroni’s or Games-Howell’s multiple comparisons test; those with non-normal distribution were presented as median (interquartile range) and analyzed with Kruskal-Wallis H test with mean rank’s *post hoc* multiple comparisons. Categorical variables were presented as n (%) and were compared using chi-square test, Pearson’s chi-square test, or Fisher’s exact test. Time-to-event variables were analyzed with Kaplan-Meier survival analyses and log-rank tests.

The differences in 3-year overall survival among the three time-weighted average blood glucose groups were analyzed using Kaplan-Meier survival analyses and log-rank tests. We used univariable Cox proportional hazard models to explore the possible association between baseline and perioperative variables and 3-year overall survival. After testing for potential collinearity with the variance inflation factor, factors with *P* < 0.20 in univariate analyses or considered clinically important were included in a multivariable Cox model to correct for confounding effects. Analyses were also performed in the subgroups of patients with or without diabetes and patients following cancer or non-cancer surgery.

A two-tailed *P* < 0.05 was considered statistically significant for each hypothesis. Bonferroni correction was made for multiple comparisons. Statistical analyses were performed with Python (version 3.7, Python Software Foundation) and SPSS 25.0 software (IBM SPSS Inc., Chicago, IL, USA).

## Results

### Participants

**FIGURE 1:**
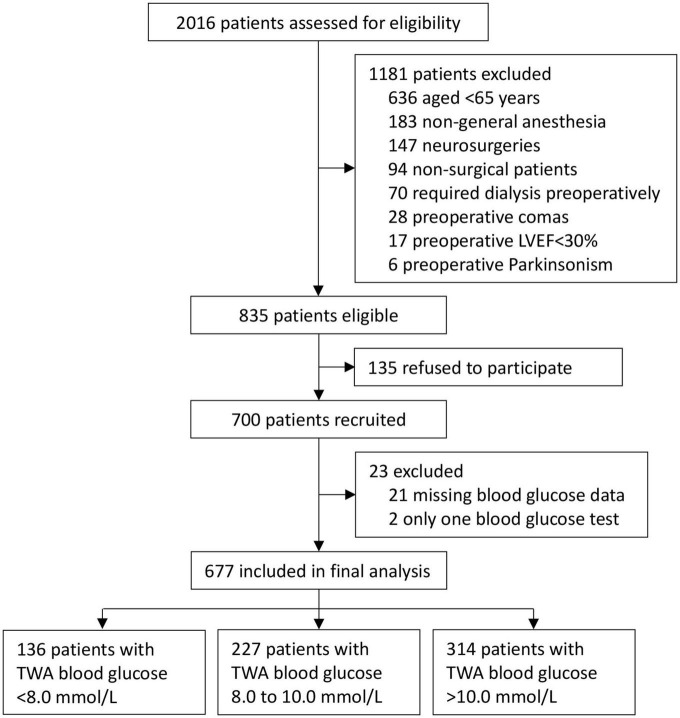
Flow chart of the study.

The underlying trial was conducted between August 17, 2011 and November 20, 2013; the 3-year follow-up was conducted between May 4, 2014 and December 13, 2016. Among 700 patients enrolled in the original study, 21 patients had missing blood glucose data and 2 had only one blood glucose test. Finally, 677 patients were included for this *post hoc* analysis. Among them, 136 (20.1%) patients had TWA blood glucose <8.0 mmol/L, 227 (33.5%) patients from 8.0 to 10.0 mmol/L, and 314 (46.4%) patients >10.0 mmol/L from ICU admission to 10 am on postoperative day 1 (Figure 1).

### Baseline and perioperative data

Regarding baseline data, patients with TWA blood glucose >10.0 mmol/L had a lower percentage of male sex, a higher body mass index, and a higher percentage of hypertension than those with TWA blood glucose <8.0 mmol/L; they also had a higher percentage and a longer duration of diabetes, received more insulin, had a higher Charlson Comorbidity Index, and had lower preoperative hemoglobin and preoperative albumin but a higher glucose than those with TWA blood glucose either <8.0 mmol/L or from 8.0 to 10.0 mmol/L. Patients with TWA blood glucose from 8.0 to 10.0 mmol/L had a higher body mass index than those with TWA blood glucose <8.0 mmol/L (Table 1).

**TABLE 1:** Baseline data.

		Time-weighted average blood glucose (mmol/L)^a^			
	All (*n* = 677)	<8.0 (*n* = 136)	8.0 to 10.0 (*n* = 227)	>10.0 (*n* = 314)	*P*-value
Age (y)	74.3 ± 6.9	73.6 ± 7.3	74.5 ± 6.7	74.5 ± 6.8	0.416
Male sex	406 (60.0%)	92 (67.6%)	145 (63.9%)	169 (53.8%)*	**0.008**
Body mass index (kg/m^2^)	23.7 ± 3.9	22.6 ± 3.8	23.8 ± 3.5*	24.2 ± 4.2*	**<0.001**
Education (y)	9 (6, 12)	9 (6, 12)	9 (6, 12)	9 (6, 12)	0.316
Preoperative comorbidities					
Stroke	157 (23.2%)	30 (22.1%)	52 (22.9%)	75 (23.9%)	0.908
COPD	41 (6.1%)	10 (7.4%)	12 (5.3%)	19 (6.1%)	0.727
Coronary heart disease	226 (33.4%)	43 (31.6%)	75 (33.0%)	108 (34.4%)	0.841
Hypertension	432 (63.8%)	71 (52.2%)	139 (61.2%)	222 (70.7%)*	**<0.001**
Arrhythmia	129 (19.1%)	31 (22.8%)	37 (16.3%)	61 (19.4%)	0.304
Type II diabetes mellitus	183 (27.0%)	12 (8.8%)	40 (17.6%)	131 (41.7%)*^†^	**<0.001**
Duration of diabetes mellitus (y)	7.0 (3.0, 15.0)	3.0 (1.5, 7.8)	4.5 (1.0, 12.0)	8.0 (4.0, 15.0)*^†^	**0.007**
Liver dysfunction^b^	19 (2.8%)	3 (2.2%)	3 (1.3%)	13 (4.1%)	0.131
Renal dysfunction^c^	32 (4.7%)	3 (2.2%)	7 (3.1%)	22 (7.0%)	**0.032**
Preoperative medication					
Hypoglycemic drugs	119 (65.0%)	6 (50.0%)	24 (60.0%)	89 (67.9%)	0.346
Insulin	78 (11.5%)	1 (0.7%)	7 (3.1%)	70 (22.3%)*^†^	**<0.001**
Beta-blockers	73 (10.8%)	12 (8.8%)	22 (9.7%)	39 (12.4%)	0.428
ACEI/ARB	122 (18.0%)	20 (14.7%)	39 (17.2%)	63 (20.1%)	0.367
Calcium channel blockers	218 (32.2%)	36 (26.5%)	71 (31.3%)	111 (35.4%)	0.169
Chronic smoking^d^	169 (25.0%)	45 (33.1%)	52 (22.9%)	72 (22.9%)	0.050
Alcoholism^e^	62 (9.2%)	20 (14.7%)	16 (7.0%)	26 (8.3%)	**0.038**
Charlson Comorbidity Index (point)^f^	3 (2, 3)	2 (2, 3)	2 (2, 3)	3 (2, 4)*^†^	**<0.001**
Preoperative ASA classification					0.178
Class II	388 (57.3%)	86 (63.2%)	121 (53.3%)	181 (57.6%)	
Class III	289 (42.7%)	50 (36.8%)	106 (46.7%)	133 (42.4%)	
Barthel Index^g^	92.1 ± 17.1	93.8 ± 14.0	92.9 ± 14.7	90.7 ± 19.7	0.138
Preoperative laboratory test					
Hemoglobin (g/L)	124.0 ± 20.5	127.8 ± 19.7	126.7 ± 19.2	120.4 ± 21.3*^†^	**<0.001**
Albumin (g/L)	38.1 ± 5.2	38.6 ± 5.2	38.8 ± 4.9	37.3 ± 5.4*^†^	**0.001**
Glucose (mmol/L)	5.6 (5.0, 6.6)	5.2 (4.8, 5.6)	5.5 (4.9, 6.1)	6.1 (5.1, 7.4)*^†^	**<0.001**
Sodium (mmol/L)	140.0 ± 3.6	139.8 ± 4.2	140.4 ± 3.0	139.8 ± 3.8	0.111
Potassium (mmol/L)	4.1 ± 0.5	4.1 ± 0.5	4.1 ± 0.4	4.1 ± 0.5	0.990
Creatinine (mmol/L)	87 (74, 102)	84 (71, 98)	87 (75, 103)	87 (74, 102)	0.237
Tumor node metastasis stage^h^					0.223
Non-cancer	130 (19.2%)	31 (22.8%)	44 (19.4%)	55 (17.5%)	
I	128 (18.9%)	25 (18.4%)	53 (23.3%)	50 (15.9%)	
II	175 (25.8%)	28 (20.6%)	59 (26.0%)	88 (28.0%)	
III	183 (27.0%)	41 (30.1%)	50 (22.0%)	92 (29.3%)	
IV	61 (9.0%)	11 (8.1%)	21 (9.3%)	29 (9.2%)	

Data are mean ± SD, n (%), or median (interquartile range). *P*-values in bold indicate <0.05. *P < 0.017 compared with patients with time-weighted average blood glucose < 8.0 mmol/L after Bonferroni correction; ^†^*P* < 0.017 compared with patients with time-weighted average blood glucose 8 to 10 mmol/L after Bonferroni correction. COPD, chronic obstructive pulmonary disease; ACEI, angiotensin converting enzyme inhibitor; ARB, angiotensin receptor blocker; ASA, American Society of Anesthesiologists.

^a^To convert to mg/dL, multiply the mmol/L value by 18.

^b^Alanine transaminase and/or aspartate transaminase more than five times the upper limit of normal.

^c^Serum creatinine >177 μmol/L.

^d^Smoking half a pack of cigarettes per day for at least 2 years.

^e^Two drinks or more daily, or weekly consumption of the equivalent of 150 mL of alcohol.

^f^According to the Charlson comorbidity index without age (26).

^g^Scores range from 0 to 100, with higher scores indicating better function (27).

^h^According to pathological results, based on the American Joint Committee on Cancer 8^th^ Edition Cancer Staging System (28).

Regarding intraoperative data, patients with TWA blood glucose >10.0 mmol/L underwent longer durations of anesthesia and surgery, received more sevoflurane and sufentanil, had more estimated blood loss, received more artificial colloid fluid infusion and blood transfusion, and endured a lower percentage of low stress surgery than those with TWA blood glucose <8.0 mmol/L (Table 2).

**TABLE 2:** Intraoperative data.

		Time-weighted average blood glucose (mmol/L)^a^			
	All (*n* = 677)	<8.0 (*n* = 136)	8.0 to 10.0 (*n* = 227)	>10.0 (*n* = 314)	*P*-value
Type of anesthesia					0.075
General	556 (82.1%)	106 (77.9%)	181 (79.7%)	269 (85.7%)	
Epidural-general	121 (17.9%)	30 (22.1%)	46 (20.3%)	45 (14.3%)	
Duration of anesthesia (min)	288 (211, 386)	269 (182, 344)	282 (213, 378)	310 (226, 417)*	**0.001**
Intraoperative medication					
Midazolam	316 (46.7%)	60 (44.1%)	97 (42.7%)	159 (50.6%)	0.153
Nitrous oxide	509 (75.2%)	100 (73.5%)	162 (71.4%)	247 (78.7%)	0.135
Sevoflurane	485 (71.6%)	85 (62.5%)	165 (72.7%)	235 (74.8%)*	**0.026**
Etomidate	259 (38.3%)	52 (38.2%)	81 (35.7%)	126 (40.1%)	0.576
Propofol	613 (90.5%)	128 (94.1%)	206 (90.7%)	279 (88.9%)	0.213
Propofol (mg)	430 (100, 1100)	520 (100, 1060)	450 (100, 1088)	300 (80, 1113)	0.424
Sufentanil (μg)	25 (10, 45)	20 (5, 35)	25 (10, 40)	30 (15, 50)*	**0.010**
Remifentanil (μg)	700 (0, 1500)	500 (0, 1200)	700 (0, 1500)	800 (0, 1600)	0.345
Dexamethasone^b^	504 (74.4%)	97 (71.3%)	170 (74.9%)	237 (75.5%)	0.639
Other glucocorticoids^c^	156 (23.0%)	35 (25.7%)	48 (21.1%)	73 (23.2%)	0.599
Intraoperative fluid balance					
Crystalloid fluid (mL)	1700 (1100, 2500)	1600 (1100, 2300)	1600 (1100, 2425)	1850 (1200, 2600)	0.076
Artificial colloid fluid (mL)	500 (500, 1000)	500 (0, 1000)	500 (500, 1000)	500 (500, 1000)*	**0.016**
Estimated blood loss (mL)	150 (50, 450)	100 (0, 250)	100 (40, 400)	200 (50, 500)*	**<0.001**
Blood transfusion^d^	161 (23.8%)	23 (16.9%)	50 (22.0%)	88 (28.0%)*	**0.029**
Urine output (mL)	450 (300, 750)	400 (300, 700)	450 (300, 700)	500 (275, 800)	0.647
Site of surgery					0.070
Genito-urinary^e^	226 (33.4%)	60 (44.1%)	73 (32.2%)	93 (29.6%)	
Gastrointestinal^f^	227 (33.5%)	38 (27.9%)	76 (33.5%)	113 (36.0%)	
Hepatobiliary-pancreatic^g^	97 (14.3%)	13 (9.6%)	32 (14.1%)	52 (16.6%)	
Lung-esophageal-thymic^h^/others^i^	127 (18.8%)	25 (18.4%)	46 (20.3%)	56 (17.8%)	
Operative Stress Score^j^					**0.002**
Low stress	52 (7.7%)	19 (14.0%)	20 (8.8%)	13 (4.1%)*	
Moderate stress	219 (32.3%)	49 (36.0%)	72 (31.7%)	98 (31.2%)	
High stress	339 (50.1%)	58 (42.6%)	119 (52.4%)	162 (51.6%)	
Very high stress	67 (9.9%)	10 (7.4%)	16 (7.0%)	41 (13.1%)	
Duration of surgery (min)	200 (126, 292)	180 (100, 255)	189 (126, 280)	218 (144, 321)*	**0.001**

Data are n (%), or median (interquartile range). *P*-values in bold indicate <0.05. *P < 0.017 compared with patients with time-weighted average blood glucose <8.0 mmol/L after Bonferroni correction.

^a^To convert to mg/dL, multiply the mmol/L value by 18.

^b^For prophylaxis of postoperative nausea and vomiting.

^c^Included methylprednisolone and hydrocortisone.

^d^Included allogeneic blood transfusion and autologous blood salvage and transfusion.

^e^Included renal, urinary bladder, ureter, and prostatic surgery.

^f^Included gastric, small intestinal, and colorectal surgery.

^g^Included liver, biliary duct, gallbladder, and pancreatic surgery.

^h^Included lung, esophageal, and malignant thymoma surgery.

^i^Included pelvic cavity, breast, thyroid, orthopedic, and larynx surgery.

^j^Stratified into five categories of physiological stress, i.e., very low stress, low stress, moderate stress, high stress, and very high stress (29).

Regarding postoperative data, patients with TWA blood glucose >10.0 mmol/L received more patient-controlled intravenous analgesia, and had higher blood glucose and coefficient of blood glucose variation than those with TWA blood glucose either <8.0 mmol/L or from 8.0 to 10.0 mmol/L; they received more blood glucose tests and developed less hypoglycemia (blood glucose ≤ 3.9 mmol/L) than those with TWA blood glucose <8.0 mmol/L; they had a higher percentage of ICU admission with intubation and received more propofol within 7 days than those with TWA blood glucose from 8.0 to 10.0 mmol/L. Patients with TWA blood glucose from 8.0 to 10.0 mmol/L had a higher blood glucose and developed less hypoglycemia than those with TWA blood glucose <8.0 mmol/L (Table 3).

**TABLE 3:** Postoperative data.

		Time-weighted average blood glucose (mmol/L)^a^			
	All (*n* = 677)	<8.0 (*n* = 136)	8.0 to 10.0 (*n* = 227)	>10.0 (*n* = 314)	*P*-value
The worst APACHE II score within 24 h	10.4 ± 3.6	10.3 ± 3.4	10.2 ± 3.3	10.6 ± 3.8	0.379
Use of patient-controlled analgesia					**0.023**
None	65 (9.6%)	19 (14.0%)	23 (10.1%)	23 (7.3%)	
Patient-controlled intravenous analgesia^b^	501 (74.0%)	93 (68.4%)	158 (69.6%)	250 (79.6%)*^†^	
Patient-controlled epidural analgesia^c^	111 (16.4%)	24 (17.6%)	46 (20.3%)	41 (13.1%)	
ICU admission with endotracheal intubation	366 (54.1%)	73 (53.7%)	107 (47.1%)	186 (59.2%)^†^	**0.020**
Time to extubation (h)^d^	5.2 (2.9, 10.9)	6.0 (2.8, 10.9)	5.0 (2.8, 10.7)	5.7 (3.0, 11.0)	0.869
Blood glucose from ICU admission to next 10 am					
Number of blood glucose tests					**0.010**
≥2 to 4 times	306 (45.2%)	72 (52.9%)	111 (48.9%)	123 (39.2%)*	
≥5 times	371 (54.8%)	64 (47.1%)	116 (51.1%)	191 (60.8%)*	
Blood glucose concentration (mmol/L)					
On admission	8.8 ± 2.7	6.9 ± 1.6	7.9 ± 1.9*	10.2 ± 2.8*^†^	**<0.001**
The highest value	12.6 ± 3.5	8.7 ± 1.4	11.1 ± 1.5*	15.2 ± 3.0*^†^	**<0.001**
The highest value, median [full range]	12.1 [6.1, 32.9]	8.7 [6.1, 16.0]	10.8 [8.4, 16.7]*	14.7 [10.4, 32.9]*^†^	**<0.001**
The lowest value	7.2 ± 1.8	5.6 ± 1.0	6.7 ± 1.1*	8.2 ± 1.8*^†^	**<0.001**
The lowest value, median [full range]	6.9 [2.4, 15.0]	5.6 [2.4, 7.3]	6.7 [3.0, 9.2]*	8.3 [3.0, 15.0]*^†^	**<0.001**
Average value	9.7 ± 2.1	7.1 ± 0.7	8.8 ± 0.6*	11.5 ± 1.6*^†^	**<0.001**
The highest minus the lowest	4.8 (3.1, 7.0)	2.8 (2.0, 3.9)	4.2 (3.0, 5.5)*	6.5 (4.8, 8.9)*^†^	**<0.001**
The highest minus admission	3.4 (1.6, 5.4)	1.7 (0.3, 2.8)	3.1 (1.3, 4.8)*	4.9 (2.9, 6.8)*^†^	**<0.001**
Time-weighted average	10.0 ± 2.2	7.2 ± 0.7	9.0 ± 0.6*	11.9 ± 1.5*^†^	**<0.001**
Time-weighted average, median [full range]	9.8 [5.3, 21.1]	7.3 [5.3, 8.0]	9.1 [8.0, 10.0]*	11.6[10.0, 21.1]*^†^	**<0.001**
Coefficient of variation (%)^e^	23.0 ± 10.3	19.4 ± 9.2	21.8 ± 9.6	25.4 ± 10.6*^†^	**<0.001**
Hypoglycemia^f^	9 (1.3%)	7 (5.1%)	1 (0.4%)*	1 (0.3%)*	**<0.001**
Severe hypoglycemia^g^	0 (0%)	0 (0%)	0 (0%)	0 (0%)	**>0.999**
Additional sedatives/analgesics within 7 days					
Midazolam	52 (7.7%)	13 (9.6%)	17 (7.5%)	22 (7.0%)	0.641
Propofol	349 (51.6%)	69 (50.7%)	100 (44.1%)	180 (57.3%)^†^	**0.009**
Morphine	198 (29.2%)	36 (26.5%)	69 (30.4%)	93 (29.6%)	0.714
Non-steroid anti-inflammatory drugs^h^	227 (33.5%)	43 (31.6%)	79 (34.8%)	105 (33.4%)	0.823
Dexmedetomidine^i^	340 (50.2%)	73 (53.7%)	116 (51.1%)	151 (48.1%)	0.524

Data are mean ± SD, n (%), or median (interquartile range). *P*-values in bold indicate <0.05. *P < 0.017 compared with patients with time-weighted average blood glucose <8.0 mmol/L after Bonferroni correction; ^†^*P* < 0.017 compared with patients with time-weighted average blood glucose from 8 to 10 mmol/L after Bonferroni correction.

APACHE II, Acute Physiology and Chronic Health Evaluation II score; ICU, intensive care unit.

^a^To convert to mg/dL, multiply the mmol/L value by 18.

^b^Established with 100 mL of 0.5 mg/mL morphine or 1.25 μg/mL sufentanil, programmed to deliver a 2 mL bolus with a lockout interval of 6-10 min and a background infusion of 1 mL/h.

^c^Established with 250 mL of 0.12% ropivacaine plus 0.5 μg/mL sufentanil, programmed to deliver a 2 mL bolus with a lockout interval of 20 min and a background infusion of 4 mL/h.

^d^Patients who were admitted to the ICU with endotracheal intubation.

^e^Calculated as the standard deviation divided by the mean glucose concentration.

^f^Defined as a blood glucose level below 3.9 mmol/L.

^g^Defined as a blood glucose level below 2.2 mmol/L.

^h^Included flurbiprofen axetil, parecoxib, aspirin-DL-lysine, and indomethacin.

^i^Administered as a continuous infusion at a rate of 0.1 μg/kg/h from ICU admission to 8 am of the next morning (23).

### Postoperative outcomes

In all patients, those with TWA blood glucose >10.0 mmol/L developed more non-delirium complications within 30 days and stayed longer in hospital than those with TWA blood glucose either <8.0 mmol/L or from 8.0 to 10.0 mmol/L; they developed more delirium than those with TWA blood glucose from 8.0 to 10.0 mmol/L. Patients with TWA blood glucose from 8.0 to 10.0 mmol/L developed less delirium than those with TWA blood glucose <8.0 mmol/L (Table 4 and Supplementary Table 1).

**TABLE 4:** Postoperative outcomes within 30 days.

		Time-weighted average blood glucose (mmol/L)^a^			
	All (n = 677)	<8.0 (*n* = 136)	8.0 to 10.0 (*n* = 227)	>10.0 (*n* = 314)	*P*-value
All patients					
Delirium within 7 days	106 (15.7%)	27 (19.9%)	23 (10.1%)*	56 (17.8%)^†^	**0.017**
Non-delirium complications within 30 days	122 (18.0%)	15 (11.0%)	33 (14.5%)	74 (23.6%)*^†^	**0.002**
Length of intensive care unit stay (h)	21 (18, 39)	22 (19, 28)	21 (17, 38)	21 (18, 40)	0.779
Length of hospital stay after surgery (d)	11 (7, 16)	10 (6, 14)	11 (7, 15)	12 (8, 19)*^†^	**<0.001**
Patients without diabetes	(*n* = 494)	(*n* = 124)	(*n* = 187)	(*n* = 183)	
Delirium within 7 days	81 (16.4%)	25 (20.2%)	19 (10.2%)*	37 (20.2%)^†^	**0.014**
Non-delirium complications within 30 days	89 (18.0%)	14 (11.3%)	29 (15.5%)	46 (25.1%)*	**0.004**
Length of intensive care unit stay (h)	21 (18, 38)	22 (18, 25)	21 (17, 40)	20 (17, 39)	0.939
Length of hospital stay after surgery (d)	11 (7, 16)	10 (6, 14)	11 (7, 15)	13 (8, 20)*^†^	**<0.001**
Patients following cancer surgery	(*n* = 547)	(*n* = 105)	(*n* = 183)	(*n* = 259)	
Delirium within 7 days	75 (13.7%)	22 (21.0%)	15 (8.2%)*	38 (14.7%)	**0.008**
Non-delirium complications within 30 days	96 (17.6%)	14 (13.3%)	24 (13.1%)	58 (22.4%)^†^	**0.019**
Length of intensive care unit stay (h)	21 (17, 37)	21 (18, 34)	21 (17, 23)	21 (17, 40)	0.579
Length of hospital stay after surgery (d)	11 (7, 16)	10 (7, 14)	10 (7, 15)	12 (8, 19)^†^	**0.012**

Data are n (%), or median (interquartile range). *P*-values in bold indicate <0.05. *P < 0.017 compared with patients with time-weighted average blood glucose <8.0 mmol/L after Bonferroni correction; ^†^*P* < 0.017 compared with patients with time-weighted average blood glucose from 8 to 10 mmol/L after Bonferroni correction.

^a^To convert to mg/dL, multiply the mmol/L value by 18.

In the subgroup of patients without diabetes, those with TWA blood glucose >10.0 mmol/L developed more non-delirium complications within 30 days and stayed longer in hospital after surgery than those with TWA blood glucose <8.0 mmol/L; they developed more delirium and stayed longer in hospital than those with TWA blood glucose from 8.0 to 10.0 mmol/L. Patients with TWA blood glucose from 8.0 to 10.0 mmol/L developed less delirium than those with TWA blood glucose <8.0 mmol/L (Table 4 and Supplementary Tables 1, 2).

In the subgroup of patients following cancer surgery, those with TWA blood glucose >10.0 mmol/L developed more non-delirium complications within 30 days and stayed longer in hospital than those with TWA blood glucose from 8.0 to 10.0 mmol/L. Patients with TWA blood glucose from 8.0 to 10.0 mmol/L developed less delirium than those with TWA blood glucose <8.0 mmol/L. In the subgroup of patients following non-cancer surgery, those with TWA blood glucose >10.0 mmol/L developed more non-delirium complications within 30 days and stayed longer in hospital than those with TWA blood glucose <8.0 mmol/L (Table 4 and Supplementary Tables 1, 2).

### Long-term outcomes

**FIGURE 2:**
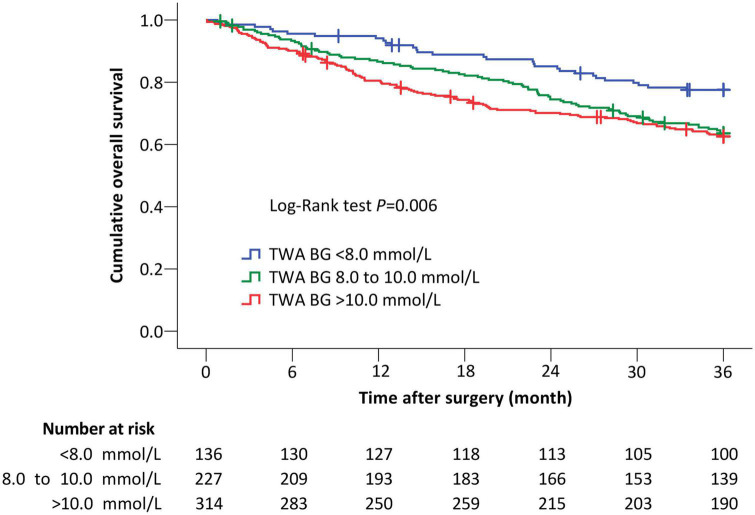
The Kaplan–Meier curves of overall survival in all patients. When compared with patients with TWA BG <8.0 mmol/L, the risk of 3-year death remained higher in those with TWA BG 8.0 to 10.0 mmol/L (adjusted HR 2.28, 95% CI 1.47 to 3.54, *P* < 0.001) and with TWA BG >10.0 mmol/L (adjusted HR 2.00, 95% CI 1.29 to 3.10, *P* = 0.002) after adjustment for confounding factors including age, sex, body mass index, chronic smoking, history of type II diabetes mellitus, American Society of Anesthesiologists classification, preoperative Barthel Index, preoperative hemoglobin, preoperative albumin, tumor-node-metastasis stage, type of anesthesia, site of surgery, Operative Stress Score, duration of surgery, intraoperative blood transfusion, endotracheal intubation on ICU admission, delirium within 7 days, and non-delirium complications within 30 days. Crosses indicate censored patients. TWA BG, time-weighted average blood glucose.

Among all patients, a total of 227 (33.5%) deaths occurred within 3 years after surgery. Specifically, there were 30 (22.1%) deaths in patients with TWA blood glucose <8.0 mmol/L, 81 (35.7%) deaths in those with TWA blood glucose from 8.0 to 10.0 mmol/L, and 116 (36.9%) deaths in those with TWA blood glucose >10.0 mmol/L. When compared with patients with TWA blood glucose <8.0 mmol/L, the unadjusted risk of 3-year death was higher in those with TWA blood glucose from 8.0 to 10.0 mmol/L (unadjusted hazard ratio [HR] 1.75, 95% CI 1.15 to 2.67, *P* = 0.009) and those with TWA blood glucose >10.0 mmol/L (unadjusted HR 1.91, 95% CI 1.28 to 2.85, *P* = 0.002). Exploratory analysis in the subgroups of patients without diabetes and patients following cancer surgery gave similar results (Figure 2, Table 5, Supplementary Figures 1, 2 and Supplementary Table 3).

**TABLE 5:** The association between time-weighted average blood glucose and three-year overall survival.

		Unadjusted^a^		Adjusted	
	All-cause deaths, n (%)	Hazard ratio (95% CI)	*P*-value	Hazard ratio (95% CI)	*P*-value
**Primary outcome**					
All patients (*N* = 677)^b^	227 (33.5%)				
TWA blood glucose <8.0 mmol/L (*n* = 136)	30 (22.1%)	Ref.		Ref.	
TWA blood glucose 8.0 to 10.0 mmol/L (*n* = 227)	81 (35.7%)	1.75 (1.15, 2.67)	**0.009**	2.28 (1.47, 3.54)	**<0.001**
TWA blood glucose >10.0 mmol/L (*n* = 314)	116 (36.9%)	1.91 (1.28, 2.85)	**0.002**	2.00 (1.29, 3.10)	**0.002**
**Exploratory analysis**					
Patients without diabetes (*N* = 494)^c^	164 (33.2%)				
TWA blood glucose <8.0 mmol/L (*n* = 124)	26 (21.0%)	Ref.		Ref.	
TWA blood glucose 8.0 to 10.0 mmol/L (*n* = 187)	66 (35.3%)	1.83 (1.16, 2.88)	**0.009**	2.55 (1.57, 4.16)	**<0.001**
TWA blood glucose >10.0 mmol/L (*n* = 183)	72 (39.3%)	2.20 (1.40, 3.44)	**0.001**	2.48 (1.53, 4.04)	**<0.001**
Patients following cancer surgery (*N* = 547)^b^	209 (38.2%)				
TWA blood glucose <8.0 mmol/L (*n* = 105)	29 (27.6%)	Ref.		Ref.	
TWA blood glucose 8.0 to 10.0 mmol/L (*n* = 183)	77 (42.1%)	1.66 (1.08, 2.54)	**0.020**	2.41 (1.54, 3.78)	**<0.001**
TWA blood glucose >10.0 mmol/L (*n* = 259)	103 (39.8%)	1.62 (1.07, 2.44)	**0.022**	1.88 (1.20, 2.95)	**0.006**

Data are n (%). *P*-values in bold indicate <0.05. Also see Supplementary Table 5. TWA, time-weighted average.

^a^Univariable Cox proportional hazard model.

^b^Cox proportional hazards models adjusted for age, sex, body mass index, chronic smoking, history of type II diabetes mellitus, American Society of Anesthesiologists classification, preoperative Barthel Index, preoperative hemoglobin, preoperative albumin, tumor-node-metastasis stage, type of anesthesia, site of surgery, Operative Stress Score, duration of surgery, intraoperative blood transfusion, endotracheal intubation on ICU admission, delirium within 7 days, and non-delirium complications within 30 days.

^c^Cox proportional hazards models adjusted for age, sex, body mass index, chronic smoking, American Society of Anesthesiologists classification, preoperative Barthel Index, preoperative hemoglobin, preoperative albumin, tumor-node-metastasis stage, type of anesthesia, site of surgery, Operative Stress Score, duration of surgery, intraoperative blood transfusion, endotracheal intubation on ICU admission, delirium within 7 days, and non-delirium complications within 30 days.

Univariable Cox regression analyses identified 24 variables with *P* < 0.20; 18 variables including history of diabetes mellitus and TWA blood glucose were included in a multivariate Cox regression analysis model. After adjustment for confounding factors, the risk of 3-year death remained higher in patients with TWA blood glucose from 8.0 to 10.0 mmol/L (adjusted HR 2.28, 95% CI 1.47 to 3.54, *P* < 0.001) and >10.0 mmol/L (adjusted HR 2.00, 95% CI 1.29 to 3.10, *P* = 0.002) than in those with TWA blood glucose <8.0 mmol/L. Exploratory analysis in the subgroups of patients without diabetes and patients following cancer surgery gave similar results (Table 5 and Supplementary Tables 3–5).

For 3-year survivors in all patients, the scores of cognitive function (assessed with the TICS-m) and four quality-of-life domains (assessed with the WHOQOL-BREF) did not differ among the three groups. Exploratory analysis in the subgroups of patients with or without diabetes and patients following cancer or non-cancer surgery gave similar results (Supplementary Table 6).

## Discussion

Our results showed that, for older patients admitted to the ICU after elective non-cardiac surgery, high early blood glucose level was associated with worse 3-year survival. Specifically, patients with TWA blood glucose ≥8.0 mmol/L had an increased risk of 3-year mortality when compared with those with TWA blood glucose <8.0 mmol/L. This association existed after adjustment for confounding factors and in the subgroups of patients without diabetes and patients following cancer surgery.

There is no commonly accepted definition of postoperative hyperglycemia (35). The adopted cutoff points varied among different studies and included 7.0 mmol/L (12, 36), 8.0 mmol/L (17, 18, 37), 10.0 mmol/L (7, 17, 37, 38), and 11.1 mmol/L (12, 17, 18, 36). For example, a large cohort study including 259,040 ICU patients showed that a mean blood glucose >8.0 mmol/L was associated with increased mortality after surgery (18). In another observational study, patients without diabetes who had blood glucose between 8.0 and 10.0 mmol/L were 1.74 times more likely to die than those with diabetes who had hyperglycemia in the same range (17). Whereas some others reported that only a hyperglycemia >10.0 mmol/L was associated with worse perioperative outcomes including 30-day mortality (7, 38). The 2012 guideline suggested that insulin therapy should be initiated when blood glucose is ≥8.3 mmol/L in general ICU patients (39); and for patients with sepsis or septic shock, recent guidelines recommended initiating insulin therapy at a blood glucose level of ≥10.0 mmol/L (40). In the present study, we adopted 8.0 mmol/L and 10.0 mmol/L as cut-off points for analysis. We also used TWA blood glucose to evaluate the blood glucose levels and to eliminate bias caused by unequal time measurements (34).

The unfavorable impact of postoperative hyperglycemia on early outcomes has been widely investigated (7, 17, 18, 38, 41). In a meta-analysis of randomized trials, tight glucose control (≤ 8.3 mmol/L) was associated improved postoperative outcomes when compared with very liberal control (upper level 10.0–12.2 mmol/L), but increased hypoglycemic events (42). In line with the above studies, our results showed that patients with severe hyperglycemia (TWA blood glucose >10 mmol/L) developed more non-delirium complications and stayed longer in the hospital after surgery; patients with moderate hyperglycemia (TWA blood glucose from 8 to 10 mmol/L) also showed slightly worse outcomes but not statistically significant. An interesting finding in our study is that patients with moderate hyperglycemia had the lowest incidence of delirium, possibly because they had a lower percentage of ICU admission with intubation. Similar results were reported in a previous trial which showed that postoperative delirium was less common in patients with routine glucose control (mean 9.5 mmol/L) than in those with tight glucose control (mean 6.6 mmol/L); the authors attributed the results to increased glucose requirements of the brain during surgical stress (43).

In clinical practice, medical stuff tends to focus on the fluctuation of blood glucose in patients with diabetes. Yet hyperglycemia in patients without diabetes might be more harmful. For example, in a recent retrospective cohort study, patients without diabetes developed more postoperative complications than those with diabetes at similar levels of hyperglycemia (44). In another study of 3026 patients following minor stroke or transient ischemic accident, those without diabetes who developed stress hyperglycemia had a higher risk of stroke recurrence within 90 days (45). Our results of subgroup analysis also showed that patients without diabetes who had severe hyperglycemia had worse postoperative outcomes; the phenomenon was not present in patients with diabetes, but possibly due to the limited sample size.

Only a few studies investigated the association between postoperative hyperglycemia and long-term outcomes. In a retrospective study, 173 patients without diabetes underwent curative gastric cancer surgery and 104 patients were included in the final analysis after propensity score matching; postoperative hyperglycemia (defined as at least one blood glucose ≥ 7.8 mmol/L within 72 h) was associated with worse 5-year overall survival and worse 5-year disease-free survival (21). In the present study, the sample size and the number of end-point events were far larger than in the above one. We therefore could provide more robust results. Our results also showed that both moderate (TWA blood glucose from 8.0 to 10.0 mmol/L) and severe (TWA blood glucose >10.0 mmol/L) hyperglycemia were associated with poor 3-year overall survival; the association existed in patients without diabetes and in those after cancer surgery. We did not find significant association between hyperglycemia and 3-year survival in patients with diabetes and in those after non-cancer surgery. A possible explanation is that the sample sizes are not enough to detect the associations in the latter two subgroups, but it is also possible that hyperglycemia is less harmful in patients with diabetes. For example, a retrospective study of 61,536 patients showed that 1-year mortality after non-cardiac surgery was lower in hyperglycemic patients with diabetes than in those without (46).

The potential mechanisms underlying our findings are not totally clear but may include the following. First, admission hyperglycemia is correlated with more severe surgical injuries and inflammation (47). Surgery-related disease and stress response might have produced harmful effects on long-term outcomes. Second, it was found that perioperative severe hyperglycemia (defined as at least one episode of blood glucose ≥ 10.0 mmol/L) attenuates T cell activation, monocytic function, and basophil count (48). The resulting immunosuppression might have produced unfavorable long-term effects. Third, hyperglycemia *per se* favors tumor cell growth (49) and thus might have promoted cancer progression. Indeed, it was reported that hyperglycemia is associated with cancer death but not non-cancer death (50), and the majority of our patients underwent cancer surgery.

The optimal perioperative glycemic level and whether targeted blood glucose control could improve long-term outcomes remain to be determined. A *post hoc* analysis of long-term survival in patients who were randomized to strict or liberal glucose control during coronary bypass surgery did not find differences between groups; however, the trial was seriously underpowered since mortality occurred in only 5.5% of 189 patients during a (mean) 40-month follow-up period (22). Many trials investigated the impact of various glycemic control strategies on 90-day mortality but did not find any benefits of tight (blood glucose 4.4 to 6.1 mmol/L) or individualized (according to glycated hemoglobin A1c level at ICU admission) glycemic control; on the contrary, tight or individualized glycemic control increased the risk of hypoglycemia and even severe hypoglycemia (51–53). Only a limited number of trials explored the short-term effects of moderate (6.1 to <7.8 mmol) glycemic control (52). According to available results, maintaining blood glucose between 6.0 and 8.0 mmol/L may be beneficial for long-term outcomes in surgical patients. Further studies are warranted in this aspect.

Strengths of our study included that the dataset came from a randomized controlled trial with large sample size and we had a high number of endpoint events. There are also some limitations. First, as with all observational studies, we could not establish a causal relationship between perioperative hyperglycemia and long-term mortality. Interventional studies are required to confirm our hypothesis. Second, preoperative hemoglobin A1c was not routinely measured. We might have overlooked some patients with unknown diabetes. Third, as a *post hoc* analysis, the sample size was not calculated for the current purpose. However, with 227 end-point events (deaths) occurred in 677 patients during a 3-year period, we have enough power to establish an association model. Forth, we only enrolled older patients admitted to the ICU after elective non-cardiac surgery in the underlying trial. This limited the generalizability of our results.

## Conclusion

We found that, in older patients admitted to the ICU after elective non-cardiac surgery, high early postoperative blood glucose (TWA blood glucose ≥ 8.0 mmol/L) was associated with worse 3-year overall survival. Future studies are required to investigate the impact of moderate glycemic control on long-term survival in this patient population.

## Data availability statement

The original contributions presented in this study are included in the article/Supplementary material, further inquiries can be directed to the corresponding authors.

## Ethics statement

The studies involving human participants were reviewed and approved by Biomedical Research Ethics Committee of the Peking University First Hospital, Beijing, China (2022 [074]). Written informed consent for participation was not required for this study in accordance with the national legislation and the institutional requirements.

## Author contributions

ML helped in data acquisition, data analysis, data interpretation, and manuscript drafting. C-MD helped in data analysis, data interpretation, and manuscript drafting. XS, D-FZ, and MD helped in data acquisition. J-HM helped in data analysis. D-XW helped in concept and design, data analysis, data interpretation, critical revision of the manuscript for important intellectual content, and supervision. All authors approved for the final version to be published.
