# Supplementary material for: Hyperglycemia is associated with worse 3-year survival in older patients admitted to the intensive care unit after non-cardiac surgery: Post hoc analysis of a randomized trial
Source: Front Med (Lausanne). 2022 Dec 12;9:1003186. doi: 10.3389/fmed.2022.1003186 (PMC9790906; doi:10.3389/fmed.2022.1003186)
Supplement: Supplementary file 1 [file Data_Sheet_1.docx]

**Supplemental Table S1. Individual complication within postoperative 30 days**

|  | **All (n=677)** | **Time-weighted average blood glucose (mmol/L)** ^a^ | | | ***P* value** |
| --- | --- | --- | --- | --- | --- |
|  |  | **<8.0 (n=136)** | **8.0 to 10.0 (n=227)** | **>10.0 (n=314)** |  |
| Cardiovascular complications, n (%) | 44 (6.5%) | 7 (5.1%) | 13 (5.7%) | 24 (7.6%) | 0.520 |
| Circulatory insufficiency ^b^ | 17 (2.5%) | 3 (2.2%) | 4 (1.8%) | 10 (3.2%) | 0.562 |
| Acute myocardial infarction ^c^ | 13 (1.9%) | 2 (1.5%) | 3 (1.3%) | 8 (2.5%) | 0.539 |
| New onset arrhythmia ^d^ | 23 (3.4%) | 2 (1.5%) | 8 (3.5%) | 13 (4.1%) | 0.354 |
| Respiratory complications, n (%) | 28 (4.1%) | 5 (3.7%) | 5 (2.2%) | 18 (5.7%) | 0.121 |
| Pulmonary infection ^e^ | 14 (2.1%) | 1 (0.7%) | 4 (1.8%) | 9 (2.9%) | 0.319 |
| Pleural effusion ^f^ | 16 (2.4%) | 4 (2.9%) | 1 (0.4%) | 11 (3.5%) | 0.061 |
| Central nervous system complications, n (%) | 107 (15.8%) | 27 (19.9%) | 23 (10.1%) * | 57 (18.2%) † | **0.015** |
| Stroke ^g^ | 3 (0.4%) | 0 (0.0%) | 0 (0.0%) | 3 (1.0%) | 0.309 |
| Delirium ^h^ | 106 (15.7%) | 27 (19.9%) | 23 (10.1%) * | 56 (17.8%) † | **0.017** |
| Urinary complications, n (%) | 21 (3.1%) | 1 (0.7%) | 6 (2.6%) | 14 (4.5%) | 0.099 |
| Acute renal failure ^i^ | 12 (1.8%) | 1 (0.7%) | 3 (1.3%) | 8 (2.5%) | 0.335 |
| Urinary tract infection ^j^ | 9 (1.3%) | 0 (0.0%) | 3 (1.3%) | 6 (1.9%) | 0.341 |
| Surgical Infection, n (%) | 20 (3.0%) | 3 (2.2%) | 7 (3.1%) | 10 (3.2%) | 0.845 |
| Wound infection ^k^ | 11 (1.6%) | 0 (0.0%) | 3 (1.3%) | 8 (2.5%) | 0.132 |
| Severe sepsis ^l^ | 9 (1.3%) | 3 (2.2%) | 4 (1.8%) | 2 (0.6%) | 0.281 |
| Other surgery-related complications, n (%) | 38 (5.6%) | 4 (2.9%) | 9 (4.0%) | 25 (8.0%) | 0.044 |
| Ileus ^m^ | 12 (1.8%) | 1 (0.7%) | 2 (0.9%) | 9 (2.9%) | 0.133 |
| Surgical bleeding ^n^ | 3 (0.4%) | 0 (0.0%) | 2 (0.9%) | 1 (0.3%) | 0.595 |
| Anastomotic leakage ^o^ | 9 (1.3%) | 1 (0.7%) | 1 (0.4%) | 7 (2.2%) | 0.178 |
| Gastrointestinal hemorrhage ^p^ | 14 (2.1%) | 3 (2.2%) | 3 (1.3%) | 8 (2.5%) | 0.608 |
| Wound dehiscence ^q^ | 5 (0.7%) | 0 (0.0%) | 2 (0.9%) | 3 (1.0%) | 0.718 |

Data are n (%). *P* values in bold indicate <0.05. * P<0.017 compared with patients with time-weighted average blood glucose <8.0 mmol/L after Bonferroni correction; † P<0.017 compared with patients with time-weighted average blood glucose from 8.0 to 10.0 mmol/L after Bonferroni correction.

^a^ To convert to mg/dL, multiply the mmol/L value by 18.

^b^ Requirement of inotropic agents or vasoconstrictors for more than 24 hours after surgery.

^c^ Concentration of cardiac troponin I exceed the diagnostic criteria for myocardial infarction as well as new Q waves (lasts for 0.03 s) or continuous (4 days) abnormal ST‐T segment.

^d^ Confirmed by 12‐lead electrocardiogram and necessitated medical treatment and/or cardioversion.

^e^ New infiltrate on chest radiograph combined with temperature over 38°C and leukocytosis.

^f^ Confirmed by chest X-ray and required thoracentesis or chest tube drainage.

^g^ Persisted new focal neurologic deficit and confirmed by neurologic imaging.

^h^ Assessed by CAM-ICU.

^i^ New onset renal failure that required renal replacement therapy.

^j^ Confirmed by urinalysis and urine culture and necessitated antibiotic therapy.

^k^ Pus expressed from the incision, and bacteria cultured from the pus.

^l^ Two or more criteria of systemic inflammatory response syndrome, with known infection and new onset dysfunction of at least one system.

^m^ Lack of bowel movement, flatulence, and requirement of intravenous fluid therapy for more than one week after surgery.

^n^ Bleeding after surgery that required secondary surgical hemostasis.

^o^ Extravasation of contrast agent in the body cavity or retroperitoneal space that required percutaneous drainage.

^p^ Decrease of hemoglobin level combined with positive gastrointestinal occult blood test results that required treatment.

^q^ Wound rupture that required secondary suturing.

**Supplemental Table S2. Postoperative outcomes within 30 days in the subgroups** **of patients with diabetes and patients following non-cancer surgery**

|  | **All (n=677)** | **Time-weighted average blood glucose (mmol/L) ^a^** | | | ***P* value** |
| --- | --- | --- | --- | --- | --- |
|  |  | **<8.0 (n=136)** | **8.0 to 10.0 (n=227)** | **>10.0 (n=314)** |  |
| Patients with diabetes | (n=183) | (n=12) | (n=40) | (n=131) |  |
| Delirium within 7 days | 25 (13.7%) | 2 (16.7%) | 4 (10.0%) | 19 (14.5%) | 0.731 |
| Non-delirium complications within 30 days | 33 (18.0%) | 1 (8.3%) | 4 (10.0%) | 28 (21.4%) | 0.174 |
| Length of intensive care unit stay (h) | 22 (18, 40) | 23 (21, 36) | 21 (17, 23) | 22 (18, 42) | 0.174 |
| Length of hospital stay after surgery (d) | 10 (7, 15) | 10 (6, 13) | 8 (6, 13) | 10 (7, 15) | 0.110 |
| Patients following non-cancer surgery | (n=130) | (n=31) | (n=44) | (n=55) |  |
| Delirium within 7 days | 31 (23.8%) | 5 (16.1%) | 8 (18.2%) | 18 (32.7%) | 0.124 |
| Non-delirium complications within 30 days | 26 (20.0%) | 1 (3.2%) | 9 (20.5%) | 16 (29.1%) * | **0.016** |
| Length of intensive care unit stay (h) | 23 (20, 43) | 23 (20, 24) | 23 (20, 44) | 22 (19, 74) | 0.573 |
| Length of hospital stay after surgery (d) | 10 (6, 15) | 8 (4, 11) | 11 (6, 14) | 12 (8, 20) * | **0.015** |

Data are n (%) or median (interquartile range). *P* values in bold indicate <0.05. * P<0.017 compared with patients with time-weighted average blood glucose <8.0 mmol/L after Bonferroni correction.

^a^ To convert to mg/dL, multiply the mmol/L value by 18.

**Supplemental Table S3. The association between time-weighted average blood glucose and three-year overall survival** **in the subgroups of patients with diabetes and patients following non-cancer surgery**

|  |  | **Unadjusted ^a^** | | **Adjusted** | |
| --- | --- | --- | --- | --- | --- |
|  | **All-cause deaths** | **Hazard ratio (95% CI)** | ***P* value** | **Hazard ratio (95% CI)** | ***P* value** |
| **Exploratory analysis** |  |  |  |  |  |
| Patients with diabetes (N=183) ^b^ | 63 (34.3%) |  |  |  |  |
| TWA blood glucose <8.0 mmol/L (n=12) | 4 (33.3%) | Ref. |  | Ref. |  |
| TWA blood glucose 8.0 to 10.0 mmol/L (n=40) | 15 (37.5%) | 1.21 (0.40, 3.66) | 0.730 | 1.75 (0.48, 6.46) | 0.399 |
| TWA blood glucose >10.0 mmol/L (n=131) | 44 (33.6%) | 1.14 (0.41, 3.18) | 0.798 | 1.12 (0.32, 3.97) | 0.862 |
| Patients following non-cancer surgery (N=130) ^c^ | 18 (13.8%) |  |  |  |  |
| TWA blood glucose <8.0 mmol/L (n=31) | 1 (3.2%) | Ref. |  | Ref. |  |
| TWA blood glucose 8.0 to 10.0 mmol/L (n=44) | 4 (9.1%) | 2.95 (0.33, 26.36) | 0.334 | 1.40 (0.40, 4.85) | 0.599 |
| TWA blood glucose >10.0 mmol/L (n=55) | 13 (23.6%) | 8.38 (1.10, 64.06) | **0.041** | 0.86 (0.26, 2.79) | 0.800 |

Data are n (%). *P* value in bold indicates <0.05.

TWA, time-weighted average.

^a^ Univariable Cox proportional hazard model.

^b^ Cox proportional hazards models adjusted for age, sex, body mass index, chronic smoking, American Society of Anesthesiologists classification, preoperative Barthel Index, preoperative hemoglobin, preoperative albumin, tumor-node-metastasis stage, type of anesthesia, site of surgery, Operative Stress Score, duration of surgery, intraoperative blood transfusion, endotracheal intubation on ICU admission, delirium within 7 days, and non-delirium complications within 30 days.

^c^ Cox proportional hazards models adjusted for age, sex, body mass index, chronic smoking, history of type II diabetes mellitus, American Society of Anesthesiologists classification, preoperative Barthel Index, preoperative hemoglobin, preoperative albumin, type of anesthesia, site of surgery, Operative Stress Score, duration of surgery, intraoperative blood transfusion, endotracheal intubation on ICU admission, delirium within 7 days, and non-delirium complications within 30 days.

**Supplemental Table S4.** **Factors in association with 3****-year overall survival (univariable analyses)**

| **Variables** | **All (n=677)** | **Hazard Ratio (95% CI)** ^a^ | ***P* value** |
| --- | --- | --- | --- |
| Age (y) | 74.3 ± 6.9 | 1.01 (0.99, 1.03) | 0.397 |
| Female sex | 271 (40.0%) | 0.68 (0.52, 0.90) | **0.007** |
| Body mass index (kg/m2) | 23.7 ± 3.9 | 0.94 (0.91, 0.97) | **<0.001** |
| Education (y) | 9 (6, 12) | 0.99 (0.96, 1.02) | 0.390 |
| Chronic smoking | 169 (25.0%) | 1.35 (1.01, 1.79) | **0.041** |
| Alcoholism | 62 (9.2%) | 1.14 (0.74, 1.76) | 0.549 |
| Type II diabetes mellitus | 183 (27.0%) | 1.01 (0.75, 1.35) | 0.955 |
| Charlson Comorbidity Index (point) | 3 (2, 3) | 1.18 (1.08, 1.28) | **<0.001** |
| Preoperative ASA classification |  |  |  |
| II | 388 (57.3%) | Ref. |  |
| III | 289 (42.7%) | 1.22 (0.94, 1.58) | **0.141** |
| Barthel Index (per 10 points) | 9.2 ± 1.7 | 0.92 (0.86, 0.99) | **0.017** |
| Preoperative hemoglobin (g/L) | 124.0 ± 20.5 | 0.99 (0.98, 0.99) | **<0.001** |
| Preoperative albumin (g/L) | 38.1 ± 5.2 | 0.96 (0.94, 0.98) | **<0.001** |
| Tumor-node-metastasis stage |  |  |  |
| Noncancer | 130 (19.2%) | Ref. |  |
| I | 128 (18.9%) | 1.31 (0.71, 2.43) | 0.391 |
| II | 175 (25.8%) | 2.19 (1.28, 3.75) | **0.004** |
| III | 183 (27.0%) | 5.13 (3.10, 8.48) | **<0.001** |
| IV | 61 (9.0%) | 7.34 (4.18, 12.9) | **<0.001** |
| Type of anesthesia |  |  |  |
| General | 556 (82.1%) | Ref. |  |
| Epidural-general | 121 (17.9%) | 1.64 (1.21, 2.21) | **0.001** |
| Duration of anesthesia (per 10 min) | 28.8 (21.1, 38.6) | 1.03 (1.02, 1.03) | **<0.001** |
| Site of surgery |  |  |  |
| Genito-urinary | 226 (33.4%) | Ref. |  |
| Gastrointestinal | 227 (33.5%) | 1.52 (1.10, 2.10) | **0.011** |
| Hepatobiliary-pancreatic | 97 (14.3%) | 1.70 (1.14, 2.52) | **0.009** |
| Lung-esophageal-thymic/others | 127 (18.8%) | 1.01 (0.66, 1.53) | 0.971 |
| Level of Operative Stress Score |  |  |  |
| Low stress | 52 (7.7%) | Ref. |  |
| Moderate stress | 219 (32.3%) | 0.96 (0.51, 1.81) | 0.900 |
| High stress | 339 (50.1%) | 1.84 (1.02, 3.33) | **0.043** |
| Very high stress | 67 (9.9%) | 3.34 (1.74, 6.41) | **<0.001** |
| Duration of surgery (per 10 min) | 20.0 (12.6, 29.2) | 1.03 (1.02, 1.04) | **<0.001** |
| Intraoperative artificial colloid (per 100 ml) | 5 (5, 10) | 1.04 (1.02, 1.06) | **<0.001** |
| Intraoperative blood transfusion | 161 (23.8%) | 1.88 (1.43, 2.48) | **<0.001** |
| Estimated blood loss (per 100 mL) | 1.5 (0.5, 4.5) | 1.02 (1.00, 1.03) | **0.002** |
| The worst APACHE II score within 24 h | 10.4 ± 3.6 | 1.05 (1.02, 1.09) | **0.002** |
| Endotracheal Intubation on ICU Admission |  |  |  |
| Without intubation | 311 (45.9%) | Ref. |  |
| With intubation | 366 (54.1%) | 1.65 (1.26, 2.17) | **<0.001** |
| Postoperative use of dexmedetomidine | 340 (50.2%) | 0.87 (0.67, 1.13) | 0.295 |
| CV of blood glucose (%) | 23.0 ± 10.3 | 1.00 (0.99, 1.02) | 0.725 |
| Time weighted average blood glucose ^b^ |  |  |  |
| <8.0 mmol/L | 30 (22.1%) | Ref. |  |
| 8.0 to 10.0 mmol/L | 81 (35.7%) | 1.75 (1.15, 2.67) | **0.009** |
| >10.0 mmol/L | 116 (36.9%) | 1.91 (1.28, 2.85) | **0.002** |
| Length of intensive care unit stay (h) | 21 (18, 39) | 1.00 (1.00, 1.01) | **0.004** |
| Delirium within 7 days | 106 (15.7%) | 1.56 (1.13, 2.16) | **0.007** |
| Non-delirium complications within 30 days | 122 (18.0%) | 1.77 (1.31, 2.39) | **<0.001** |
| Length of hospital stay after surgery (d) | 11 (7, 16) | 1.02 (1.01, 1.02) | **<0.001** |

Data are mean ± SD, n (%), or median (interquartile range). *P* values in bold indicate <0.20.

ASA, American Society of Anesthesiologists

^a^ Univariable Cox proportional hazard model.

^b^ To convert to mg/dL, multiply the mmol/L value by 18.**Supplemental Table S5. Association between predictors and 3-year overall survival**

| **Variables** | **Univariate analysis** ^a^ | | **Multivariate analysis ^b^** | |
| --- | --- | --- | --- | --- |
|  | **Hazard Ratio (95% CI)** | ***P* value** | **Hazard Ratio (95% CI)** | ***P* value** |
| Age (y) | 1.01 (0.99, 1.03) | 0.397 | 1.02 (1.00, 1.04) | 0.101 |
| Female sex | 0.68 (0.52, 0.90) | **0.007** | 0.72 (0.52, 1.00) | **0.048** |
| Body mass index (kg/m2) | 0.94 (0.91, 0.97) | **<0.001** | 0.94 (0.90, 0.98) | **0.003** |
| Chronic smoking | 1.35 (1.01, 1.79) | **0.041** | 1.14 (0.82, 1.58) | 0.449 |
| Type II diabetes mellitus | 1.01 (0.75, 1.35) | 0.955 | 1.04 (0.76, 1.44) | 0.794 |
| Preoperative ASA classification |  |  |  |  |
| II | Ref. |  | Ref. |  |
| III | 1.22 (0.94, 1.58) | **0.141** | 1.51 (1.14, 2.00) | **0.004** |
| Barthel Index (per 10 points) | 0.92 (0.86, 0.99) | **0.017** | 0.88 (0.81, 0.96) | **0.003** |
| Preoperative hemoglobin (g/L) | 0.99 (0.98, 0.99) | **<0.001** | 0.99 (0.99, 1.00) | 0.133 |
| Preoperative albumin (g/L) | 0.96 (0.94, 0.98) | **<0.001** | 1.00 (0.97, 1.03) | 0.869 |
| Tumor-node-metastasis stage |  |  |  |  |
| Noncancer | Ref. |  | Ref. |  |
| I | 1.31 (0.71, 2.43) | 0.391 | 2.01 (1.01, 4.01) | 0.048 |
| II | 2.19 (1.28, 3.75) | **0.004** | 2.21 (1.17, 4.16) | **0.014** |
| III | 5.13 (3.10, 8.48) | **<0.001** | 7.58 (4.12, 13.96) | **<0.001** |
| IV | 7.34 (4.18, 12.9) | **<0.001** | 7.52 (3.89, 14.54) | **<0.001** |
| Type of anesthesia |  |  |  |  |
| General | Ref. |  | Ref. |  |
| Epidural-general | 1.64 (1.21, 2.21) | **0.001** | 1.41 (1.01, 1.97) | **0.046** |
| Site of surgery |  |  |  |  |
| Genito-urinary | Ref. |  | Ref. |  |
| Gastrointestinal | 1.52 (1.10, 2.10) | **0.011** | 1.06 (0.73, 1.52) | 0.772 |
| Hepatobiliary-pancreatic | 1.70 (1.14, 2.52) | **0.009** | 1.92 (1.14, 3.23) | **0.014** |
| Lung-esophageal-thymic/others | 1.01 (0.66, 1.53) | 0.971 | 1.17 (0.71, 1.93) | 0.545 |
| Operative Stress Score |  |  |  |  |
| Low stress | Ref. |  | Ref. |  |
| Moderate stress | 0.96 (0.51, 1.81) | 0.900 | 0.58 (0.29, 1.16) | 0.120 |
| High stress | 1.84 (1.02, 3.33) | **0.043** | 0.70 (0.36, 1.35) | 0.284 |
| Very high stress | 3.34 (1.74, 6.41) | **<0.001** | 0.90 (0.40, 2.06) | 0.809 |
| Duration of surgery (per 10 min) | 1.03 (1.02, 1.04) | **<0.001** | 1.01 (1.00, 1.03) | **0.042** |
| Intraoperative blood transfusion | 1.88 (1.43, 2.48) | **<0.001** | 1.12 (0.81, 1.54) | 0.492 |
| Endotracheal intubation on ICU admission |  |  |  |  |
| Without intubation | Ref. |  | Ref. |  |
| With intubation | 1.65 (1.26, 2.17) | **<0.001** | 1.35 (1.00, 1.82) | 0.050 |
| Time-weighted average blood glucose ^c^ |  |  |  |  |
| <8.0 mmol/L | Ref. |  | Ref. |  |
| 8.0 to 10.0 mmol/L | 1.75 (1.15, 2.67) | **0.009** | 2.28 (1.47, 3.54) | **<0.001** |
| >10.0 mmol/L | 1.91 (1.28, 2.85) | **0.002** | 2.00 (1.29, 3.10) | **0.002** |
| Delirium within 7 days | 1.56 (1.13, 2.16) | **0.007** | 1.56 (1.08, 2.24) | **0.017** |
| Non-delirium complications within 30 days | 1.77 (1.31, 2.39) | **<0.001** | 1.21 (0.87, 1.69) | 0.255 |

*P* values in bold indicate <0.05.

ASA, American Society of Anesthesiologists.

^a^ Univariable Cox proportional hazard model.

^b^ Factors with P<0.20 or were considered clinically important were entered into the multivariable Cox proportional hazard model. Charlson Comorbidity Index was not included because of correlation with American Society of Anesthesiologists classification and Tumor-node-metastasis stage; duration of anesthesia and intraoperative artificial colloid were not included because of correlation with duration of surgery; estimated blood loss was not included because of correlation with intraoperative blood transfusion; the worst APACHE II score within 24 h was not included because of correlation with American Society of Anesthesiologists classification, Operative Stress Score, and endotracheal intubation on ICU admission; length of intensive care unit stay and length of hospital stay after surgery were not included because of correlation with delirium within 7 days and non-delirium complications within 30 days.

^c^ To convert to mg/dL, multiply the mmol/L value by 18.

**Supplemental Table S6. Cognitive function and quality of life in 3-year survivors**

|  | **All (n=677)** | **Time-weighted average blood glucose (mmol/L) ^a^** | | | ***P* value** |
| --- | --- | --- | --- | --- | --- |
|  |  | **<8.0 (n=136)** | **8.0 to 10.0 (n=227)** | **>10.0 (n=314)** |  |
| **Secondary outcomes** |  |  |  |  |  |
| 3-year survivors in all patients | (n/N=422/667) | (n/N=96/136) | (n/N=138/227) | (n/N=188/314) |  |
| Cognitive function (point) ^b^ | 36.3 ± 5.4 | 36.6 ± 5.2 | 36.2 ± 6.3 | 36.1 ± 4.9 | 0.835 |
| Quality of life (point) ^c^ |  |  |  |  |  |
| Physical | 71.4 ± 17.4 | 71.8 ± 17.8 | 72.3 ± 18.6 | 70.5 ± 16.4 | 0.617 |
| Psychological | 74.9 ± 16.6 | 75.6 ± 15.9 | 74.8 ± 18.1 | 74.6 ± 16.0 | 0.889 |
| Environment | 75.7 ± 14.4 | 74.9 ± 14.4 | 76.5 ± 16.0 | 75.5 ± 13.2 | 0.728 |
| Social relationship | 71.6 ± 14.5 | 71.9 ± 14.8 | 72.0 ± 14.6 | 71.1 ± 14.2 | 0.824 |
| **Exploratory analysis** |  |  |  |  |  |
| 3-year survivors in patients without diabetes | (n/N=310/494) | (n/N=89/124) | (n/N=114/187) | (n/N=107/183) |  |
| Cognitive function (point) ^b^ | 36.1 ± 5.5 | 36.6 ± 5.3 | 35.9 ± 6.3 | 36.1 ± 4.8 | 0.674 |
| Quality of life (point) ^c^ |  |  |  |  |  |
| Physical | 71.2 ± 17.4 | 72.2 ± 18.3 | 71.0 ± 18.8 | 70.7 ± 15.2 | 0.827 |
| Psychological | 74.5 ± 16.6 | 75.7 ± 16.3 | 73.6 ± 18.3 | 74.5 ± 14.8 | 0.651 |
| Environment | 75.2 ± 14.3 | 75.4 ± 14.5 | 75.2 ± 15.8 | 75.2 ± 12.4 | 0.996 |
| Social relationship | 71.3 ± 14.4 | 72.4 ± 14.8 | 71.3 ± 14.4 | 70.5 ± 14.2 | 0.658 |
| 3-year survivors in patients with diabetes | (n/N=112/183) | (n/N=7/12) | (n/N=24/40) | (n/N=81/131) |  |
| Cognitive function (point) ^b^ | 36.6 ± 5.3 | 36.4 ± 4.2 | 38.0 ± 6.4 | 36.3 ± 5.1 | 0.390 |
| Quality of life (point) ^c^ |  |  |  |  |  |
| Physical | 71.7 ± 17.5 | 66.8 ± 10.9 | 78.4 ± 16.6 | 70.2 ± 17.9 | 0.096 |
| Psychological | 75.8 ± 16.9 | 73.2 ± 9.9 | 80.6 ± 16.3 | 74.6 ± 17.5 | 0.299 |
| Environment | 76.9 ± 14.8 | 68.8 ± 13.3 | 82.3 ± 15.8 | 76.0 ± 14.2 | 0.059 |
| Social relationship | 72.3 ± 14.6 | 65.5 ± 14.0 | 75.7 ± 15.3 | 71.9 ± 14.3 | 0.238 |
| 3-year survivors in patients following cancer surgery | (n/N=316/547) | (n/N=67/105) | (n/N=100/183) | (n/N=149/259) |  |
| Cognitive function (point) ^b^ | 36.9 ± 4.8 | 37.4 ± 4.6 | 36.7 ± 5.3 | 36.8 ± 4.6 | 0.645 |
| Quality of life (point) ^c^ |  |  |  |  |  |
| Physical | 72.6 ± 16.2 | 73.4 ± 16.8 | 72.6 ± 16.8 | 72.2 ± 15.7 | 0.885 |
| Psychological | 76.0 ± 15.6 | 77.4 ± 15.0 | 74.7 ± 16.2 | 76.3 ± 15.4 | 0.507 |
| Environment | 77.1 ± 13.5 | 76.7 ± 14.0 | 76.9 ± 14.7 | 77.3 ± 12.6 | 0.957 |
| Social relationship | 72.4 ± 13.9 | 73.1 ± 13.7 | 72.9 ± 14.0 | 71.8 ± 13.9 | 0.745 |
| 3-year survivors in patients following non-cancer surgery | (n/N=106/130) | (n/N=29/31) | (n/N=38/44) | (n/N=39/55) |  |
| Cognitive function (point) ^b^ | 34.4 ± 6.7 | 34.7 ± 5.9 | 34.9 ± 8.3 | 33.8 ± 5.4 | 0.740 |
| Quality of life (point) ^c^ |  |  |  |  |  |
| Physical | 67.7 ± 20.2 | 68.0 ± 19.8 | 71.5 ± 22.8 | 63.7 ± 17.5 | 0.241 |
| Psychological | 71.3 ± 19.1 | 71.3 ± 17.2 | 75.1 ± 22.6 | 67.7 ± 16.4 | 0.241 |
| Environment | 71.6 ± 16.1 | 70.6 ± 14.7 | 75.2 ± 19.2 | 68.9 ± 13.4 | 0.218 |
| Social relationship | 69.0 ± 15.9 | 69.0 ± 16.9 | 69.7 ± 16.0 | 68.4 ± 15.3 | 0.933 |

Data are mean ± SD.

^a^ To convert to mg/dL, multiply the mmol/L value by 18.

^b^ Assessed with the Telephone Interview for Cognitive Status-modified; a 12-item questionnaire that provides an assessment of global cognitive function by verbal communication via telephone, score ranges from 0 to 50, with higher score indicating better function.

^c^ Assessed with the World Health Organization Quality of Life-Brief version; a 24-item questionnaire that provides assessments of the quality of life in physical, psychological, social relationship, and environmental domains; for each domain, the score ranges from 0 to 100, with higher score indicating better function.
