# Supplementary material for: Hyperglycemia is associated with worse 3-year survival in older patients admitted to the intensive care unit after non-cardiac surgery: Post hoc analysis of a randomized trial
Source: Front Med (Lausanne). 2022 Dec 12;9:1003186. doi: 10.3389/fmed.2022.1003186 (PMC9790906; doi:10.3389/fmed.2022.1003186)
Supplement: Supplementary file 3 [file Image_2.pdf]

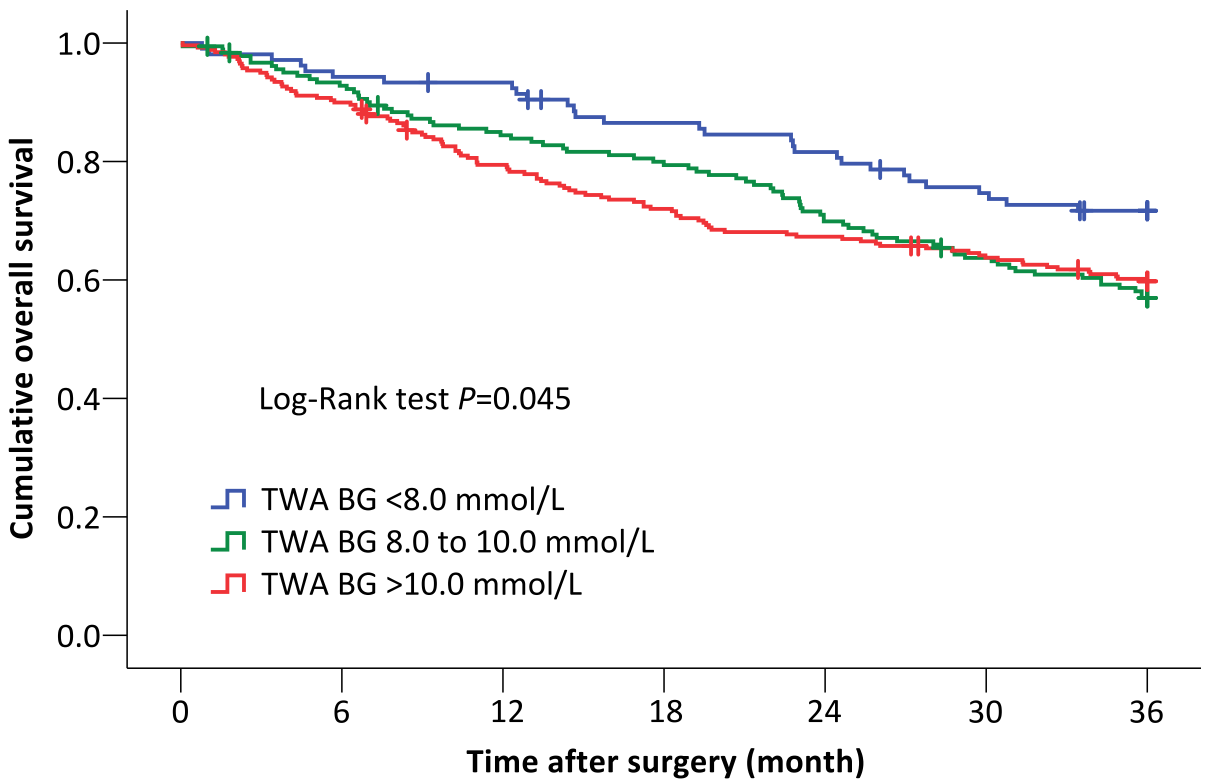

Number at risk

Number at risk	0	6	12	18	24	30	36
<8.0 mmol/L	105	99	97	88	83	75	70
8.0 to 10.0 mmol/L	183	167	151	142	125	113	101
>10.0 mmol/L	259	233	203	184	172	161	151

Supplemental Figure S2. The Kaplan-Meier curves of overall survival in patients following cancer surgery. When compared with patients with TWA BG <8.0 mmol/L, the risk of 3-year mortality was higher in those with TWA BG from 8.0 to 10.0 mmol/L (adjusted hazard ratio 2.41, 95% CI 1.54 to 3.78, P<0.001) and in those with TWA BG >10.0 mmol/L (adjusted hazard ratio 1.88, 95% CI 1.20 to 2.95, P=0.006) after adjustment for confounding factors including age, sex, body mass index, chronic smoking, American Society of Anesthesiologists classification, history of diabetes mellitus, preoperative Barthel Index, preoperative hemoglobin, preoperative albumin, tumor-node-metastasis stage, type of anesthesia, site of surgery, Operative Stress Score, duration of surgery, intraoperative blood transfusion, endotracheal intubation on ICU admission, delirium within 7 days, and non-delirium complications within 30 days. Crosses indicate censored patients. TWA BG, time-weighted average blood glucose.
